# Effects of Adipose Tissue-Specific Knockout of Delta-like Non-Canonical Notch Ligand 1 on Lipid Metabolism in Mice

**DOI:** 10.3390/ijms25010132

**Published:** 2023-12-21

**Authors:** Xin Lu, Xibi Fang, Jiaqi Mi, Yue Liu, Ruimin Liu, Guanghui Li, Yue Li, Runjun Yang

**Affiliations:** 1 College of Animal Sciences, Jilin University, Changchun 130062, China; luxin20@mails.jlu.edu.cn (X.L.); fangxibi@jlu.edu.cn (X.F.); mijq18@mails.jlu.edu.cn (J.M.); yueliu22@mails.jlu.edu.cn (Y.L.); ghli21@mails.jlu.edu.cn (G.L.); li_yue22@mails.jlu.edu.cn (Y.L.); 2 College of Animal Science and Veterinary Medicine, Tianjin Agricultural University, Tianjin 300392, China; liri0122@163.com

**Keywords:** *DLK 1*, adipose tissue-specific knockout mice, triglyceride, lipid metabolism

## Abstract

Delta-like non-canonical Notch ligand 1 (*DLK1*), which inhibits the differentiation of precursor adipocytes, is a recognized marker gene for precursor adipocytes. Lipids play a crucial role in energy storage and metabolism as a vital determinant of beef quality. In this study, we investigated the mechanism of the *DLK1* gene in lipid metabolism by constructing adipose tissue-specific knockout mice. We examined some phenotypic traits, including body weight, liver coefficient, fat index, the content of triglyceride (TG) and cholesterol (CHOL) in abdominal white adipose tissue (WAT) and blood. Subsequently, the fatty acid content and genes related to lipid metabolism expression were detected in *DLK1*^−/−^ and wild-type mice via GC-MS/MS analysis and quantitative real-time PCR (qRT-PCR), respectively. The results illustrated that *DLK1*^−/−^ mice exhibited significant abdominal fat deposition compared to wild-type mice. HE staining and immunohistochemistry (IHC) results showed that the white adipocytes of *DLK1*^−/−^ mice were larger, and the protein expression level of *DLK1*^−/−^ was significantly lower. Regarding the blood biochemical parameters of female mice, *DLK1*^−/−^ mice had a strikingly higher triglyceride content (*p* < 0.001). The fatty acid content in *DLK1*^−/−^ mice was generally reduced. There was a significant reduction in the expression levels of the majority of genes that play a crucial role in lipid metabolism. This study reveals the molecular regulatory mechanism of fat metabolism in mice and provides a molecular basis and reference for the future application of the *DLK1* gene in the breeding of beef cattle with an excellent meat quality traits. It also provides a molecular basis for unravelling the complex and subtle relationship between adipose tissue and health.

## 1. Introduction

Lipids play a crucial role in energy storage and metabolism and serve as essential signaling molecules for numerous cellular activities [1]. Animal lipid metabolism is mainly affected by genetic, nutritional and environmental factors, and there are differences in lipid metabolism and genetic mechanisms between different species. Fat content directly impacts the tenderness, flavor and nutritional value of beef. Fat accumulation between muscles can enhance the marbling grade and palatability of beef [2]. Fat deposition in muscle involves a series of intricate processes, including preadipocytes proliferation and differentiation as well as lipid metabolism, which are regulated by the relevant functional genes [3]. The fatty acid composition of adipose tissue is closely related to meat quality [4]. Therefore, it is of great importance to investigate the molecular mechanisms underlying adipogenesis and the regulatory pathways governing genes associated with meat quality traits. The adipogenesis process can be divided into two main stages: the differentiation from mesenchymal stem cells to preadipocytes, and the differentiation from preadipocytes to adipocytes [5]. Throughout the rearing of beef cattle, adipocytes will continue to expand, accumulate fat in distinct positions and simultaneously undergo proliferation and differentiation in tandem with the growth process.

Delta-like non-canonical Notch ligand 1 (*DLK1*), also known as preadipocyte factor 1 (*Pref*-*1*), is a transmembrane protein that contains epidermal growth factor (EGF)-like repeats that are homologous to the Notch/Delta/Serrate family [6,7]. *DLK1* is an imprinted gene primarily expressed from the paternal chromosome. It plays a vital role in regulating fetal growth and development [8]. Animal and in vitro studies have demonstrated that *DLK1* is a gatekeeper for adipogenesis by preventing adipocyte differentiation [9,10]. *DLK1* inhibits adipocyte differentiation in adipose tissue and contributes to the process of skeletal muscle regeneration [11]. A previous study showed that *DLK1* could switch nutrient metabolism towards fatty acid oxidation, particularly during the transition from birth to weaning in genetically modified mice [12,13,14]. Indeed, *DLK1*-null mice exhibit accelerated weight gain and hyperlipidemia in adulthood, supporting their anti-adipogenic actions [15]. Research conducted on cattle has indicated that *DLK1* has the ability to suppress adipocyte differentiation, while *DLK1* intervention promotes adipogenesis, implying that *DLK1* plays a crucial role in maintaining the state of preadipocytes [16]. Furthermore, studies have revealed that *DLK1* knockout can suppress the expression of the myogenic regulatory transcription factor *MyoD*, thereby facilitating the self-renewal of activated satellite cells [17]. Moreover, it has been observed that the bovine *DLK1*-*DIO3* locus is significantly associated with milk, carcass, fertility and health traits [18]. Accurate reprogramming of the *DLK1*-*DIO3* imprinted region is also crucial for the methylation regulation in cloned neonatal pigs [19]. In beef cattle, our previous study indicated that *DLK1* can affect the TG content by constructing interference and overexpression vectors. Meanwhile, two single nucleotide polymorphisms (SNPs), IVS3 + 478 C > T and IVS3 + 609 T > G, were greatly associated with the carcass fat coverage rate, loin eye muscle area, and fat color score. Therefore, *DLK1* may serve as potential genetic markers for future meat quality traits in beef cattle breeding [20].

Candidate genes associated with meat quality traits have undergone extensive validation in the livestock industry to acquire enhanced economic attributes. However, beef cattle are unsuitable as model animals due to their protracted growth cycle, sluggish reproduction, and exorbitant feeding costs. Conversely, mice, being one of the classic model animals, possess the advantages of rapid reproduction, abbreviated growth cycle and economical feeding requirements [21]. These experimental animals offer an exceptional model for investigating disease mechanisms, screening drug efficacy and conducting functional analysis of key trait candidate genes. Therefore, transgenic animal models can be employed to explore lipid metabolism effectively. Conditional knockouts were selected because homozygous knockouts of the systemic *DLK1* gene result in embryonic or neonatal death. Hence, the *DLK1* adipose tissue-specific knockout mouse model was generated utilizing the CRISPR/Cas9 combined with the Cre-loxP system. Wild-type mice were used as the control.

Based on the previous functional verification of *DLK1* in lipid differentiation at the population and cellular level, this study further substantiated its function in the lipid metabolism of *DLK1* adipose tissue-specific knockout mice in vivo. Through monitoring the growth curve of mice, assessing fat and liver weight, examining TG and CHOL content in WAT and analyzing a diverse array of plasma biochemistry index, as well as evaluating the fatty acid composition in adipose tissue and the expression of genes involved in lipid metabolism, we elucidated the impact of the *DLK1* gene on the intricate process lipid metabolism during the dynamic period of growth and development in vivo. These findings provided valuable insights into the role of *DLK1* in the molecular breeding of beef cattle and would be useful for further research on meat quality breeds.

## 2. Results

### 2.1. The Identification of Adipose Tissue-Specific Knockout of DLK1 Mice (DLK1^−/−^ Mice)

**Figure 1:**
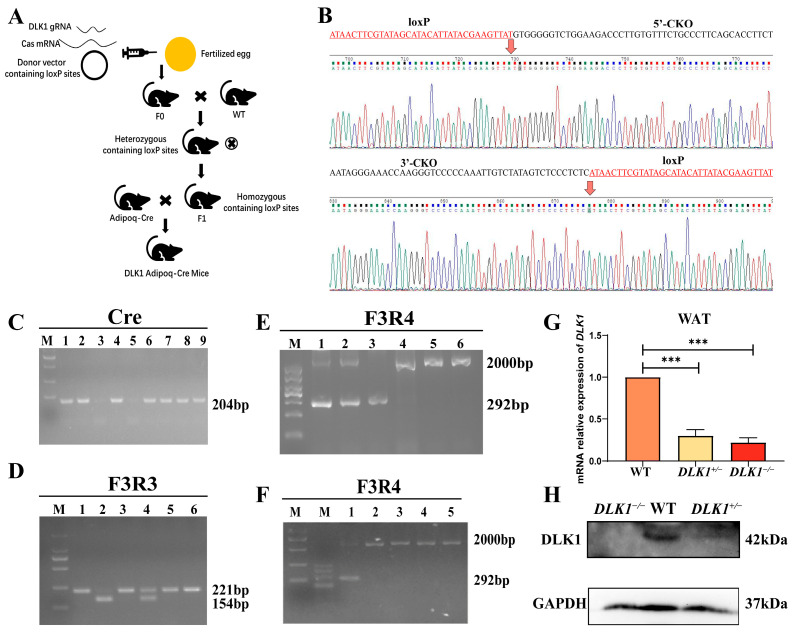
The identification result of *DLK*1^−/−^ mice. (**A**) Preparation flow chart of *DLK1* adipose tissue-specific knockout mice. (**B**) Sanger sequencing results of PCR-amplified fragments from *DLK1* loxP locus knockout mice. (**C**) Gel electrophoresis results of Cre PCR products. (**D**) Gel electrophoresis results of PCR products for loxP site from tail-tip tissue DNA. (**E**) Gel electrophoresis results of PCR products of F3R4 from WAT. (**F**) Gel electrophoresis results of PCR products of F3R4 from WAT, hearts, livers, spleens, lungs, and kidneys. (**G**) Expression level of *DLK1* in WAT, *** *p* < 0.001. (**H**) Western blot results of DLK1 in WAT.

The construct methods of *DLK1* adipose tissue-specific knockout mice are shown in a brief flow chart (Figure 1A). The loxP sites were inserted on both sides of the third exon of *DLK1*. When the Cre enzyme activity was positive in mice, the loxP site could be cut to inactivate *DLK1*. Homozygous adipose tissue-specific knockout mice (*DLK1*^−/−^ mice) were generated when the gene was knocked out on both chromosomes. The Sanger sequencing results of the loxP site are shown in Figure 1B. The agarose gel electrophoresis results are expressed in Figure 1C–F. Mice without Cre activity were considered wild-type, as shown in lanes 3, 5 in Figure 1C. When the loxP site was successfully inserted into the exon of the *DLK1* gene, the PCR product length was 221 bp, and when the loxP site was not inserted into the exon of *DLK1* gene, the PCR product length was 154 bp. Mice with a 221 bp and 154 bp fragment and positive Cre were considered heterozygous (*DLK1*^+/−^), as shown in lane 4 in Figure 1D. Mice with a 221 bp fragment of the F3R3 product and positive Cre were considered homozygous (*DLK1*^−/−^), as shown in lanes 1, 3, 5, 6 in Figure 1D. Furthermore, the knockout of *DLK1* in WAT was confirmed using DNA from WAT. When the Cre gene recognized the loxP site on *DLK1* gene and successfully cleaved, the PCR product lengths were 292 bp, as shown in lane 3 in Figure 1E. When the PCR product lengths were 292 bp and 2000 bp, the genotype was identified as heterozygous (*DLK1*^+/−^). When the Cre gene was inactivated, the *DLK1* gene was not cut. The length of the PCR product was 2000 bp, as shown in lanes 4, 5, 6 in Figure 1E. The negative controls from other tissues (hearts, livers, spleens, lungs, and kidneys) were also included (Figure 1F). qRT-PCR and Western blot were used to assess the expression of *DLK1*, which was significantly lower in homozygous mice than wild-type mice (Figure 1G,H).

### 2.2. Effects on Growth and Development Traits in DLK1^−/−^ Mice

**Figure 2:**
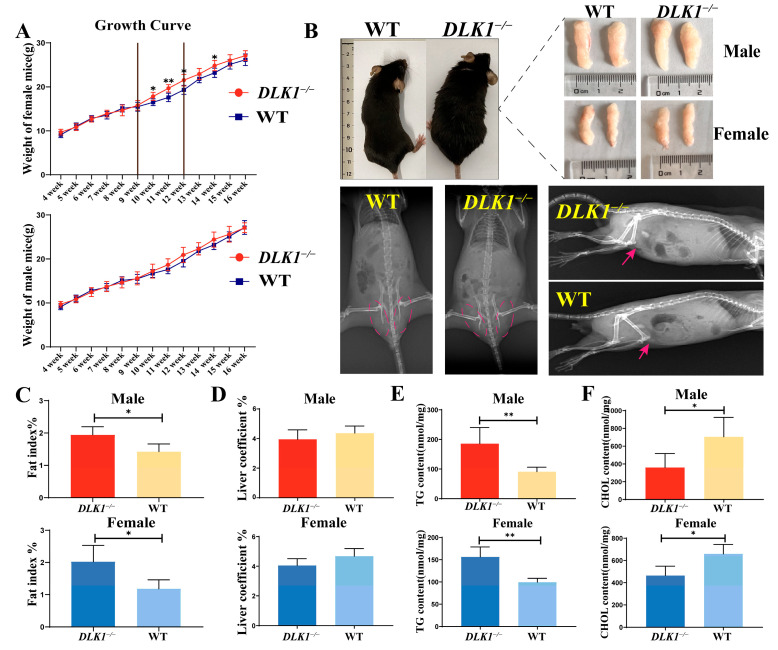
Effects on growth and development traits in *DLK1*^−/−^ mice. (**A**) Growth curve of both female and male mice (* *p* < 0.05, ** *p* < 0.01). (**B**) Comparison of abdominal white fat in homozygous and wild-type mice. The two upper X-ray images are positioned in the dorsoventral orientation, and the subsequent two are situated on the left side. Red arrows and dashed lines represent the location of abdominal white fat in mice. (**C**) Fat index of homozygous and wild-type mice. (**D**) Liver coefficient of homozygous and wild-type mice. (**E**) Triglycerides content in WAT of homozygous and wild-type mice. (**F**) Cholesterol content in WAT of homozygous and wild-type mice.

In this study, the effects of *DLK1* knockout on weight gain were investigated. We monitored changes in body weight gain between homozygous and wild-type mice at 4–16 weeks old. The results indicated that the body weight gain of homozygous female mice did not significantly differ from wild-type mice until 9 weeks of age, while from 10 weeks onwards, the body weight gain of homozygous mice significantly increased (*p* < 0.05) until 14 weeks (*n* ≥ 6). However, the body weight gain of homozygous male mice did not exhibit a considerable difference (*p* > 0.05, Figure 2A). In addition, whole-body photographs and X-ray results also showed higher fat content in homozygous mice than wild-type mice (Figure 2B). Meanwhile, homozygous males showed a slight increase in body weight compared with wild-type males. To compare the difference more intuitively in fat deposition levels between *DLK1*^−/−^ mice and wild-type mice under normal feeding conditions, we observed and weighed the fat tissue of 16-week-old mice. The fat coefficient and fat tissue weight of *DLK1*^−/−^ mice were also significantly higher than those of wild-type mice, indicating increased fat deposition (Figure 2C). The liver coefficient exhibited a slight decrease in homozygous mice compared to wild-type mice; nevertheless, it was not considered significant (Figure 2D). Additionally, the triglyceride content in white adipose tissue was significantly up-regulated in homozygous female mice compared to wild-type mice, while the cholesterol content was slightly elevated (Figure 2E,F).

### 2.3. Examination of Tissue Morphology in DLK1^−/−^ Mice

**Figure 3:**
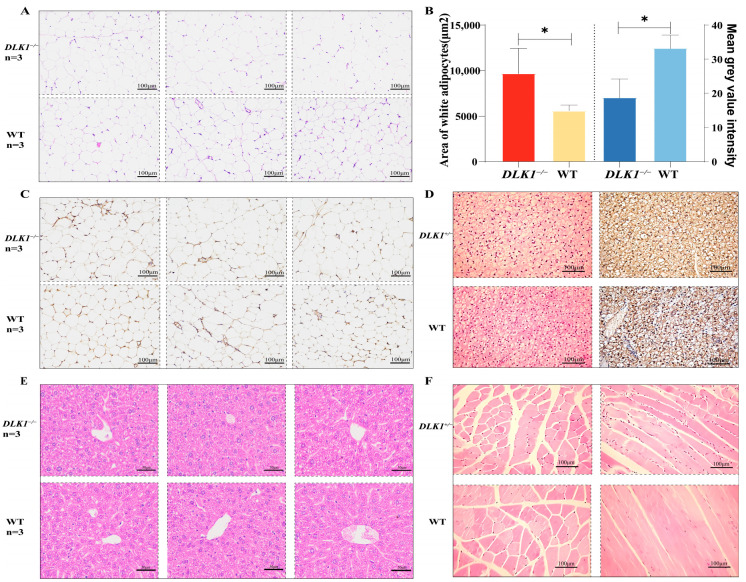
Examination of tissue morphology in *DLK1*^−/−^ mice. (**A**) HE results in WAT of wild-type and *DLK1*^−/*−*^ mice. (**B**) Quantification of adipocyte size (**left**) and IHC positive area mean gray value intensity (**right**), * *p* < 0.05. (**C**) IHC results of WAT in wild-type and *DLK1*^−/−^ mice. (**D**) HE and IHC results in BAT of wild-type genotype and DLK1^+/−^ mice. (**E**) HE and IHC results in muscle of wild-type and *DLK1*^+/−^ mice. (**F**) HE results in the liver of wild-type and *DLK1*^−/−^ mice.

The WAT of wild-type and homozygous mice at the age of 22 weeks was collected from the same batch for HE staining and immunohistochemistry (IHC). Histological analyses of the abdominal WAT showed that the area of white adipocytes in homozygous mice was significantly larger than that in wild-type mice (Figure 3A). Quantification of the adipocyte area using ImageJ software (ImageJ 1.53t, National Institutes of Health, USA, Java 11.0.7) also showed that the area of white adipocytes in homozygous mice was considerably increased compared with wild-type mice (*p* < 0.05, Figure 3B). The results of positive intensity in IHC sections and quantification positive area mean gray value intensity as determined in ImageJ software confirmed the reduced protein expression of *DLK1* in the abdominal WAT of homozygous mice (*p* < 0.05, Figure 3B,C). This consequence indicated that the protein expression levels of *DLK1* were significantly reduced in the abdominal white adipose tissue of homozygous mice. In addition, the results of HE staining and IHC of brown adipose tissue (BAT) (Figure 3D) manifested that there was no significant difference between heterozygous and wild-type mice. The area of hepatocyte in mice was not evident in different genotypes (Figure 3E). HE staining of leg muscle showed that there were more intramuscular fat deposits in *DLK1*^+/−^ mice than WT mice (Figure 3F).

### 2.4. Detection of Blood Biochemistry Index in DLK1^−/−^ Mice

**Figure 4:**
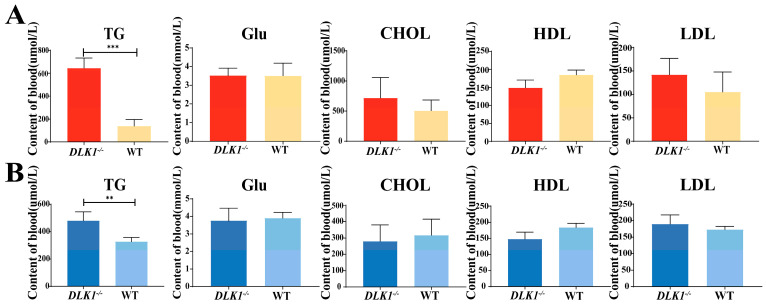
Blood biochemistry index in *DLK1*^−/−^ mice and wild-type mice. (**A**) TG, Glu, CHOL, HDL, LDL contents in plasma of female homozygous and wild-type mice. (**B**) TG, Glu, CHOL, HDL, LDL contents in plasma of male homozygous and wild-type mice ** *p* < 0.01, *** *p* < 0.001.

Homozygous and wild-type mice were selected for eyeball blood collection at 22 weeks of age. After centrifugation at 4 °C, the plasma was collected and its biochemical parameters were analyzed. Female *DLK1*^−/−^ mice had a strikingly higher triglyceride content than their wild-type counterparts (*p* < 0.001). Additionally, there was a slight decrease in blood glucose (Glu) levels. In contrast, the cholesterol content, as well as the levels of high-density lipoprotein (HDL) and low-density lipoprotein (LDL), exhibited a slight increase (Figure 4A). Similar results were obtained in male mice, where the triglyceride content was significantly higher in homozygous mice than wild-type mice (*p* < 0.01). Moreover, the glucose content in the blood of male homozygous mice decreased, while CHOL, HDL and LDL content decreased (Figure 4B). These findings regarding triglyceride levels in the blood were consistent with the observations made in WAT. The results indicated that in both female and male mice, the specific knockout of *DLK1* in adipose tissue led to an increase in triglyceride content in lipid metabolism. However, *DLK1* knockout did not have any significant impact on the glucose, cholesterol, HDL and LDL levels in the blood.

### 2.5. Effect of Tissue-Specific Knockout of DLK1 on Fatty Acids Content in White Adipose Tissue

**Figure 5:**
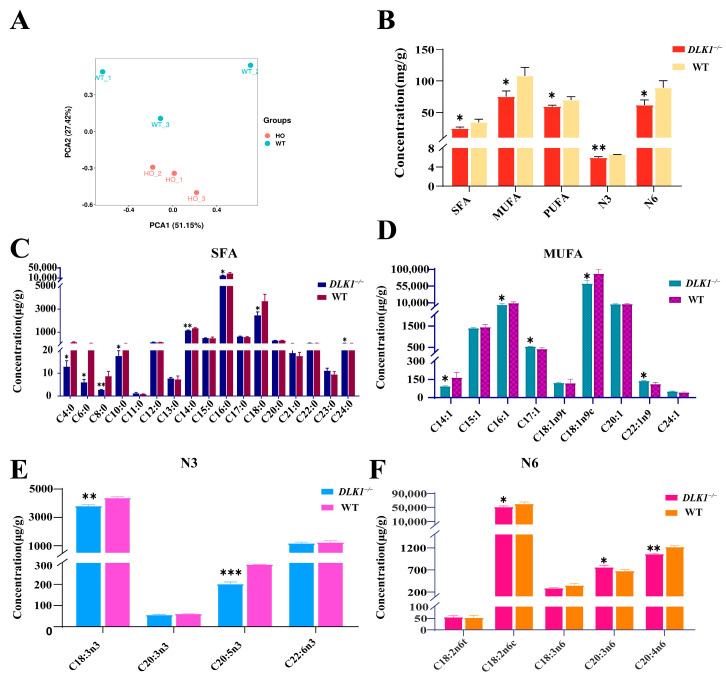
Fatty acids composition in the WAT of mice and wild-type mice. (**A**) PCA clustering results. (**B**) Total intracellular content of various fatty acids in WAT. (**C**) Saturated fatty acids at high levels in WAT. (**D**) Monounsaturated fatty acid with high levels in WAT. (**E**,**F**) Higher levels of polyunsaturated fatty acids (N:3, N:6) in WAT of different genotypes.* *p* < 0.05, ** *p* < 0.01, *** *p* < 0.001.

To further investigate the impact of *DLK1* on lipid metabolism, fatty acids were extracted from the abdominal WAT of wild-type mice and *DLK1* adipose tissue knockout mice, respectively. The principal component analysis (PCA) demonstrated that the fatty acids content varied across different groups (Figure 5A). The results revealed that the fatty acid content in the *DLK1*^−/−^ mice was generally reduced, which might be related to the reduced triglycerides hydrolysis. When the *DLK1* gene was knocked out in WAT, saturated fatty acids (SFAs), monounsaturated fatty acid (MUFAs) and polyunsaturated fatty acid (PUFAs) were significantly decreased (*p* < 0.05). N-6 PUFA was significantly decreased (*p* < 0.05), while N-3 exhibited a large decrease (*p* < 0.01) (Figure 5B). Among the SFAs, the myristate (C14:0), palmitate (C16:0) and stearate (C18:0) contents were higher, and the myristate content in the homozygous group was considerably lower than that in the wild-type group (*p* < 0.01) (Figure 5C). Oleate (C18:1n9c) had the highest content among the MUFAs. Compared with the wild-type group, the myristoleate (C14:1), palmitoleate (C16:1) and oleate contents in WAT of the homozygous group were significantly reduced, but the 10-Heptadecenoate (C17:1) and erucate (C22:1n9) contents were significantly increased (Figure 5D). Among the PUFAs, the highest contents were linked to α-linolenate (C18:3n3) and methyl linoleate (C18:2n6c). The α-linolenate (C18:3n3), eicosapentaenoate (C20:5n3), methyl linoleate (C18:2n6c) and arachidonate (C20:4n6) contents were significantly lower than those of wild-type mice. The homogamma linolenate (C20:3n6) content was significantly higher than that of wild-type mice (*p* < 0.05) (Figure 5E,F). In general, the fatty acid contents in knockout mice were mostly reduced, which might indicate a reduction in triglycerides hydrolysis.

### 2.6. Differentially Expressed Genes Related to Lipid Metabolism in WAT of DLK1^−/−^ Mice

**Figure 6:**
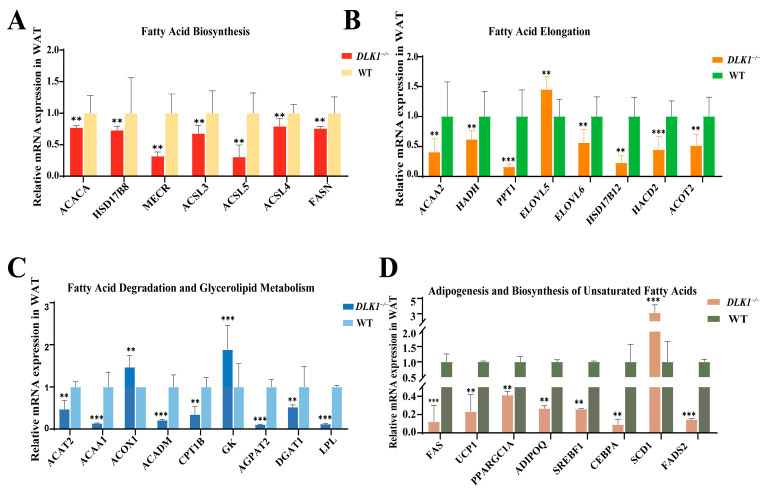
Relative expression of genes associated with lipid metabolism in WAT. (**A**) Differentially expressed genes related to fatty acid biosynthesis in WAT of *DLK1*^−/−^ Mice. (**B**) Differentially expressed genes related to fatty acid elongation in WAT of *DLK1*^−/−^ Mice. (**C**) Differentially expressed genes related to fatty acid degradation and glycerolipid metabolism in WAT of *DLK1*^−/−^ Mice. (**D**) Differentially expressed genes related to thermogenesis, adipogenesis and biosynthesis of unsaturated fatty acids in WAT of *DLK1*^−/−^ Mice. ** *p* < 0.01, *** *p* < 0.001.

To further elucidate the impact of *DLK1* on adipose tissue deposition in mice, the expression levels of genes involved in lipid metabolism were analyzed in testicular appendage lipids from either homozygous or wild-type 22 week-old male mice (*n* = 3) by qRT-PCR. The results showed that the mRNA expression levels of *Acetyl*-*CoA Carboxylase* (*ACACA*), *17β*-*Hydroxysteroid dehydrogenases* (*HSD178B*), *mitochondrial trans*-*2*-*enoyl*-*CoA reductase* (*MECR*), *Acyl-CoA Synthetase Long Chain Family Member 3* (*ACSL3*), *Acyl*-*CoA Synthetase Long Chain Family Member 4* (*ACSL4*), *Acyl*-*CoA Synthetase Long Chain Family Member 5* (*ACSL5*) and *Fatty Acid Synthase* (*FASN*) genes, which are involved in the fatty acid biosynthesis pathway, were significantly decreased in the *DLK1*^−/−^ group compared with the wild-type group (*p* < 0.01, Figure 6A). Meanwhile, fatty acid elongation related genes (*Acetyl*-*CoA Acyltransferase 2* (*ACAA2*), *Hydroxyacyl*-*CoA Dehydrogenase* (*HADH*), *Palmitoyl*-*Protein Thioesterase 1* (*PPT1*), *ELOVL Fatty Acid Elongase 6* (*ELOVL6*), *Hydroxysteroid 17*-*Beta Dehydrogenase 12* (*HSD17B12*), 3-Hydroxyacyl-CoA Dehydratase 2 (*HACD2*), *Acyl*-*CoA Thioesterase 2* (*ACOT2*)) were significantly reduced, except for *ELOVL Fatty Acid Elongase 5* (*ELOVL5*) (*p* < 0.01, Figure 6B). *Acetyl*-*CoA Acetyltransferase 2* (*ACAT2*), *Acetyl*-*CoA Acyltransferase 1* (*ACAA1*), *Acyl*-*CoA Dehydrogenase Medium Chain* (*ACADM*) and *Carnitine Palmitoyltransferase 1B* (*CPT1B*) genes, related to the fatty acid decomposition pathway were significantly down-regulated, while the *Acyl*-*CoA Oxidase 1* (*ACOX1*) gene significantly increased. *1*-*Acylglycerol*-*3*-*Phosphate O*-*Acyltransferase 2* (*AGPAT2*), *Diacylglycerol O-Acyltransferase 1* (*DGAT1*) and *Lipoprotein Lipase* (*LPL*) genes, related to the triglyceride metabolism pathway, were significantly decreased, while *Glycerol kinase* (*GK*) genes were significantly increased (*p* < 0.01, Figure 6C). The genes associated with thermogenesis (*Uncoupling Protein 1* (*UCP1*) and *PPARG Coactivator 1 Alpha* (*PPARGC1A*)) and adipogenesis (*Adiponectin, C1Q And Collagen Domain Containing* (*ADIPOQ*), *Sterol Regulatory Element Binding Transcription Factor 1* (*SREBF1*) and *CCAAT Enhancer Binding Protein Alpha* (*CEBPA*)) were significantly down-regulated. However, genes associated with unsaturated fatty acid production, *Stearoyl*-*CoA desaturase 1* (*SCD1*), were significantly up-regulated while the *FADS2* gene was significantly down-regulated (*p* < 0.01, Figure 6D). The qRT-PCR results demonstrate a significant reduction in the expression levels of the majority of genes that play a crucial role in lipid metabolism when the *DLK1* gene was knocked out.

## 3. Discussion

The Adipoq-cre mouse is a genetically stable and widely utilized tool mouse. In their report, Farrar et al. highlighted that the Adipoq-cre enzyme can selectively express itself in adipose tissue, generating the Cre enzyme and thereby achieving adipocyte-specific gene modification [22,23]. Previous identification of mice genotypes has posed considerable challenges, with the PCR results of the Cre gene presenting indistinct outcomes, leading to only a small homozygotes population being accurately identified. By adjusting the PCR conditions of the Cre gene, the extension time was increased to 1 min and the number of cycles was adjusted to 38, ensuring unequivocal identification of homozygous individuals. The efficiency of Cre-loxP-mediated recombination decreases with increasing genetic distance when the two endpoints are located on the same chromosome. However, this efficiency is not limiting even when the genetic distance is maximized [24]. Nevertheless, further verification is needed to ascertain whether loxP can be excised after Adipoq-Cre expression. Consequently, additional examination of mRNA and protein levels in adipose tissue is indispensable to determining the specific absence of *DLK1* in WAT.

Genomic imprinting, through DNA methylation, results in the differential expression of imprinted genes from either the maternally or paternally inherited chromosomes [25]. In mammals, imprinting is restricted to certain loci regulated by cis-acting elements called imprinting control regions (ICRs) [26]. The expression of *DLK1* was found exclusively on the paternal allele due to differential methylation and the maternal allele was not expressed [27]. The imprinted *DLK1*-*DIO3* locus has biological significance in gestational progression, during which the occurrence of imprinting errors leads to developmental disorders [28]. Li et al. conducted studies on the imprinting status and methylation regulation of *DLK1*-*DIO3* and found that it was imprinted and observed in dead cloned pigs [19]. During this experiment, four mice with muscle rigidity were found; they had the smallest body weight in the litter, extremely slow growth, stiff limbs and no body fat deposition, which are thought to be related to the complex regulatory mechanism of the *DLK1-DIO3* locus. Since homozygous mice are difficult to obtain in the early stage, they were obtained by mating with heterozygous mice throughout the experiment. Thus, the mechanism of paternally imprinted genes in lipid metabolism was not further explored, which could be a direction of subsequent research.

Although the disparity in body weight between homozygous and wild mice was not remarkably significant, there was a substantial disparity in the TG content in WAT. Genetic ablation of *DLK1* in the myogenic lineage resulted in a reduced body weight and skeletal muscle mass due to reductions in myofiber numbers and myosin heavy chain IIB gene expression [17]. Differences were also observed in both liver and WAT coefficients. This partially indicates that the weight of the liver, a pivotal organ in lipid metabolism, varies across different genotypes of mice. The abdominal adipose tissue also exhibits dissimilarities, indicating that inhibition of the *DLK1* gene can promote the generation of mature adipocytes. Additionally, it was observed that the sizes of *DLK1*^−/−^ adipocytes were larger and the immunofluorescence intensity of the *DLK1*^−/−^ group was weaker in WAT sections, further confirming that *DLK1* is involved in the inhibition of preadipocyte differentiation into mature adipocytes. Likewise, researchers have discovered that *DLK1* in its membrane-bound inhibited preadipocyte proliferation by regulating several key components into the G1/S phase [29,30].

Furthermore, HE staining and IHC were conducted on BAT, liver and muscle tissues, revealing that adipose tissue-specific knockout had no significant impact on other tissues. This serves as evidence that our mouse model was constructed perfectly. The blood biochemical index values confirm that *DLK1* knockout significantly reduces the TG content, but had no significant effect on the blood glucose, LDL and HDL contents. This result indicates that the model animals were in a healthy state, and *DLK1* knockout had no effect on their health of mice, but only affected the function of lipid metabolism. Hence, the *DLK1* gene can be used as a molecular marker for beef cattle molecular breeding in the future.

The fatty acid contents of the knockout mice were mostly reduced, which might indicate a reduction in the hydrolysis of triglycerides. SFAs may affect cardiovascular risk through non-lipoprotein mechanisms [31]. PUFAs, as advantageous components of fatty acids, possess diverse biological properties and can lower the prevalence of cardiovascular and cerebrovascular diseases, as well as prevent obesity [32]. The qRT-PCR results demonstrate that there is a significant reduction in the expression levels of the majority of genes that play a crucial role in lipid metabolism when the *DLK1* gene is knocked out. However, *ELOVL5*, *GK* and *SCD1* expression increased in the homozygous group. *ELOVL5* is involved in MUFAs (C16, C18) and PUFAs (C18, C20, C22), regulating the metabolism of lipids and carbohydrates in the liver and thus affecting the TG and glucose concentration [33]. *SCD1* is a desaturase that catalyzes the dehydrogenation of SFAs into MUFAs. It mainly acts on palmitic acid (C16:0) and stearic acid (C18:0) in SFAs catalyzed to palmitoleic acid (C16:1) and oleic acid (C18:1) in MUFAs [34,35]. *GK* is a rate-limiting enzyme necessary for the utilization of free glycerol in cells, which can catalyze the phosphorylation of free glycerol into glycerol 3-phosphate [36]. On the one hand, glycerol 3-phosphate can be used as an important raw material for lipid synthesis, and it can participate in the synthesis of glycerides. On the other hand, glycerol 3-phosphate is also be closely linked to glucose metabolism [37] and can participate in metabolic processes such as gluconeogenesis and decomposition for energy supply [38,39].

The role of *DLK1* in regeneration is still controversial. Since mesenchymal progenitor cells, also known as adipocyte progenitor cells, only express *DLK1* during muscle regeneration, it has been reported that mice overexpressing *DLK1* exhibit an increased muscle mass while *DLK1*-knockout mice exhibit a reduced muscle mass. This result indicates that *DLK1* is a key regulator of skeletal muscle mass during development [40]. The process of muscle regeneration shares some features with that of muscle development.

This discovery holds immense significance for comprehending the intricate role of the *DLK1* gene in lipid metabolism and sheds light on the underlying mechanism that contribute to obesity without significant weight gain but with increased fat mass. This is also consistent with our results of reduced expression levels of genes involved in lipolysis. By understanding the correlation between the deletion of the *DLK1* gene and an impaired lipid metabolism, researchers can gain valuable insights into potential therapeutic targets and strategies to combat obesity. Moreover, this finding enhances our understanding of the complex interplay between genetics and fat deposition, providing a foundation for further investigations into the molecular pathways involved in fat deposition and metabolism. By utilizing *DLK1* as a genetic marker, breeders can select animals with desirable meat quality traits at an early stage, accelerating the breeding progress and ensuring the production of high-quality meat production.

## 4. Materials and Methods

### 4.1. Preparation of Adipose Tissue-Specific Knockout Mice (DLK1^−/−^ Mice)

The preparation flow chart of *DLK1* adipose tissue-specific knockout mice is shown in Figure 1A. Briefly, the gRNA to the *DLK1* gene, the donor vector with loxP sites and Cas9 mRNA were co-injected into fertilized C57BL/6 eggs to generate targeted conditional knockout offspring. C57BL/6 of F0-generation mice was obtained from Cyagen Biosciences, Suzhou. Founder animals of the F0-generation were identified by PCR, followed by sequence analysis and bred with wild-type mice to test germline transmission and F1 animal generation. Subsequently, heterozygous mice were inter-crossed to produce homozygous mice with the loxP site. Then, homozygous mice were bred with a tissue-specific Cre^+^-deleted mouse to create mice that were heterozygous for the targeted allele and heterozygous for the Cre transgene. Afterwards, heterozygous mice were crossed with homozygous mice. Approximately 25% of the offspring should be homozygous mice with a Cre transgene. Mice were combined in a ratio of 1:1 with 3 males and females. The ear tag numbers, genotype and date of combination of both sire and dam were recorded. After the first day of mating, females were observed for the formation of vaginal plugs and isolated in separate cages. The time of birth and the number of neonatal mice were also recorded. All the mice were raised in the specific pathogen-free (SPF) animal breeding environment at the Animal Experimental Center of Jilin University with an individually ventilated caging (IVC) system. The feeding temperature was 22–26 °C, and the relative humidity was 40–60%. All the mice were leisurely fed, and the living environment was quiet and peaceful. The weight of the mice was recorded from the 4th to the 13th week after birth, and the growth curve was drawn.

### 4.2. Identification of Mice Genotypes

Genomic DNA was extracted from tail tissues of neonatal mice aged 35 days using the TIANamp Genomic DNA kit (Tiangen, Beijing, China), strictly following the instructions. The homozygous loxP site was verified by PCR with mmu-*DLK1*-F3 and R3 primer. The presence of the Cre gene was identified by PCR using universal primers of Cre-F and Cre-R. The adipose tissue DNA was extracted for PCR in order to further confirm the knockout effect of *DLK1* with F3 and R4 primers. The primer sequences are shown in Table 1. The PCR amplification was conducted using a 20 µL system containing a combination of 10 µL of green Taq mix (Vazyme, Nanjing, China), 5 µmole in 0.5 µL of each primer, 50 ng/µL in 3 µL of mouse genomic DNA and 6 µL of RNase-Free ddH_2_O. The PCR amplification conditions were as follows: the PCR mixture was initially incubated at 95 °C for 5 min, followed by 35 cycles consisting of denaturation at 95 °C for 30 s, annealing at 56 °C for 40 s for the homozygous loxP fragment and annealing at 53 °C for 60 s for the Cre gene and the extension step was performed at 72 °C for 1 min. A final extension was performed at 72 °C for 10 min. The PCR products were detected by 3% agarose gel electrophoresis.

### 4.3. Analysis of Triglyceride and Cholesterol Contents in Adipose Mice Tissue

TG and cholesterol was detected by the Tissue/Cell Triglyceride (TG) Content Enzymatic Determination Kit and Enzymatic assay kit to determine the total cholesterol (CHOL) in tissue cells (E1013, E1015, Applygen Technologies Inc., Beijing, China) according to the manufacturer’s instructions. Adipose tissue was added into lysate, grinded by electric homogenizer and left on ice for 10 min. A total of 10 µL of the liquid was removed for protein concentration assay correction. The remaining liquid was heated at 70 °C for 10 min. The upper clarified liquid was centrifuged at 2000 rpm for 5 min at room temperature and removed for enzymatic assay. The optical density of each sample was determined using a multi-function microplate reader (Biotech, San Francisco, CA, USA). The experiment was repeated three times and three technical replicates were performed for each sample. The total protein concentration was measured with the Enhanced BCA Protein Quantitation Assay Kit (KGP902, KeyGEN BioTECH, Nanjing, China). The TG content was finally corrected per mg protein content.

### 4.4. Tissue HE Staining and Immunohistochemistry

White adipose, brown adipose and liver tissues were collected from 5-month-old *DLK1^−/−^* and wild-type mice. The tissues were washed in saline and placed in 4% paraformaldehyde fixative for dehydration and transparency. Then, they were placed in an embedding box after trimming and immersed in 80% alcohol for 45 min, 90% alcohol for 30 min, 95% alcohol for 30 min, 100% alcohol for 30 min, 100% alcohol for 45 min, xylene for 20 min and xylene for 20 min sequentially. The tissue block was immersed in a wax solution at the melting point of 58 °C for 20 min. This was repeated three times, and then the wax-soaked tissue was embedded. The surface of the tissue block was corrected with a sharp microtome, and the complete tissue section with a thickness of about 5 μm was slowly cut off. The tissue was spread out in a 42 °C water bath without folds, and then flattened on a slide. The tissue slices were cut and placed in a 60 °C oven for baking for 2 h. Stain sections with Hematoxylin solution for 3–5 min, then rinsed with tap water. Then, treat the section with Hematoxylin Differentiation solution, and rinsed with tap water. The section was treated with Hematoxylin Scott Tap Bluing agent and rinsed with tap water. 85% ethanol for 5 min and 95% ethanol for 5 min. Finally, the stain sections were stained with Eosin dye for 5 min. Then, the section was observed under the microscope (Nikon TE2000, Tokyo, Japan).

The IHC procedure was different from that of HE staining. After dewaxing the paraffin sections, they were placed in a 3% hydrogen peroxide solution and incubated at room temperature in the dark for 25 min. The sections were washed 3 times by shaking in PBS in a decolorization shaker for 5 min each time. The tissue was covered by dropping 3% BSA uniformly in the histochemical circle and blocked for 30 min at room temperature. The blocking solution was gently removed, rabbit polyclonal anti-DLK1 antibody (1:1000 dilution in 1 × Tris-buffered saline, bs-4556R, Bioss Antibodies, Beijing, China) was added dropwise to the sections and the sections were placed flat in a wet box and incubated overnight at 4 °C. The plates were washed three times and incubated with sheep anti-rabbit HRP antibody 1:200 (GB23301, Servicebio, Wuhan, China) for 50 min at room temperature. DAB color solution (G1212, Servicebio, Wuhan, China) was added and the color development time was controlled under the microscope, where the positive color was brown-yellow. The slices were observed via a microscope (Nikon TE2000, Tokyo, Japan) for image acquisition and analysis.

### 4.5. Assay of Blood Biochemical Indices in Mice

The same batch of *DLK1*^−/−^ and wild-type mice were fasted for 12 h under free-drinking conditions. The next day, blood samples were collected from the posterior orbital venous plexus in 1.5 mL anticoagulant tubes containing potassium citrate and then centrifuged for 20 min at 3000 rpm at 4 °C. The supernatant was collected in a new 1.5 mL sterilized centrifuge tube for subsequent determination of plasma biochemical parameters. Plasma TG, CHOL, Glu, HDL and LDL levels were detected by Enzymic assay kit for total triglyceride, cholesterol, glucose, HDL, LDL in liquid samples (E1003, E1005, E1010, E1018 and E1017, Applygen Technologies, Beijing, China), which were used as described in the instructions. Then, the absorbance of the samples was detected using a multi-function microplate reader (Biotech, San Francisco, CA, USA).

### 4.6. Analysis of Genes Related to Lipid Metabolism Expression by Quantitative Real-Time PCR

Total RNA of white adipose tissue was extracted with RNAiso Plus reagent (Takara, Dalian, China). cDNA was synthesized using a reverse transcription kit (Tiangen, Beijing, China), 2 µg of total RNA, 4 µL of 5 × FastKing-RT SuperMix and RNase-Free ddH_2_O was added to make the total volume up to 20 µL. The cDNA synthesis conditions were as follows: incubate at 42 °C for 15 min, and at 95 °C for 3 min. qRT-PCR was conducted through with SuperReal PreMix Plus (SYBR Green) (Tiangen, Beijing, China) utilizing the specific primers shown in Table 2. qRT-PCR was performed in a 10 µL of reaction medium with 0.25 µL of forward and reverse primer, 5 µL of 2 × SuperReal PreMix Plus, 1 µL of cDNA and 3.5 µL of ddH_2_O. The following procedure was used: 95 °C for 15 min and 40 cycles of 95 °C for 10 s and 60 °C for 30 s in a PCRmax (Eco, Staffordshire, UK). Technical and biological replicates were performed three times and *β*-*actin* was used as an internal standard to normalize the mRNA expression level using the 2^−∆∆CT^ method.

### 4.7. Fatty Acid Detection in Mouse Adipose Tissue

An appropriate amount of samples was weighed and added into a 2.0 mL EP tube, and diethyl ether was added. The mixture was shocked and centrifuged, and then the supernatant was extracted twice. It was then combined with two times the amount of extraction solution, dried with nitrogen, and n-hexane was added to redissolve them. After the NaOH-methanol solution was ossified, 14% BF_3_-methanol was added to methyl ester, vortically mixed into saturated NaCl solution. The solution was centrifuged (500 r/min, 5 min) and the upper layer was taken into the vial for GC-MS/MS detection and analysis.

### 4.8. Western Blot

The total protein concentration in white adipose tissue was determined using radioimmunoprecipitation assay (RIPA) buffer (AR0102, Boster, Wuhan, China) following the reagent specifications. The protein concentration was determined via an enhanced bicinchoninic acid protein quantitation assay (Keygen Biotech, Nanjing, China) using a spectrophotometer (Biotech, San Francisco, CA, USA). Proteins were resolved using SDS-PAGE and transferred onto a polyvinylidene difluoride membrane (ISEQ00005, Immobilon-PSQ, Burlington, MA, USA). Immunoblotting was performed with DLK1 antibodies at a 1:1000 dilution (bs-4556R, Bioss Antibodies, Beijing, China). The immunoblots were developed using a BeyoECL Plus Kit (P0018S, Beyotome, Shanghai, China), and the signal intensities were captured by a Tanon 5200 chemiluminescence/fluorescence image analysis system (Tanon 5200 Multi, Shanghai, China).

### 4.9. Statistical Analysis

Experimental data are displayed as Mean ± SEM. The *p*-value less than 0.05 has been defined as a statistically significant difference. The comparative Ct method (2^−ΔΔCt^) was used for qRT-PCR data on relative gene expression. GraphPad Prism 9 software (GraphPad, San Diego, CA, USA) was used in the data analysis with a two-tailed *t*-test.

## 5. Conclusions

In conclusion, in this study, we have shown that the adipose tissue-specific knockout of *DLK1* significantly increased fat deposition and TG contents and decreased the contents of most fatty acids in the mice by creating conditional knockout mice, monitoring body weight, detecting lipid metabolism-related biochemical indicators such as TG and CHOL in blood and adipose tissue, and detecting differential fatty acids and genes. This coincided with the lipid metabolism gene results. This study provides a molecular basis and reference for the future application of the *DLK1* gene in the breeding of beef cattle with excellent meat quality traits and human disease.

## Figures and Tables

**Table 1:** Primer sequences for the identification of *DLK1* adipose tissue-specific knockout.

Primers	Sequences (5′–3′)
Mmu-*DLK1*-F3	CACTGTAGATCACTGTACCAACTG
Mmu-*DLK1*-R3	CATTTGGTGGTCTTTGTTGAGTCC
Mmu-*DLK1*-R4	TTAGAATGGAGGGGTCCTACCCTA
A-Cre-F	GAACGCACTGATTTCGACCA
A-Cre-R	GCTAACCAGCGTTTTCGTTC

**Table 2:** qRT-PCR primer of genes related to lipid metabolism.

Primers	Forward Sequences (5′–3′)	Reverse Sequences (5′–3′)
*DLK1*	TGTGACAAGTGTGTAACTGCC	TTCCATTGTTGGCGCAG
*β*-*actin*	GCACCACACCTTCTACAA	TACGACCAGAGGCATACA
*ACACA*	GCTATGGAAGTCGGCTATGGA	TTGTCAGGAAGAGGCGGATG
*HSD17B8*	GTCGTGGCTCCATCATCAAC	GCAGTTACCTTGTCCTTCACTT
*MECR*	CGATTGGCTCTCAACTGTGTT	TCATCTGGACTGTGGTTCTTCT
*ACSL3*	GCGAGAAGGATTCCAAGACTG	TGTGGTGAAGAGTAGCCGATT
*ACSL5*	AAGTTGGCACAAGGAGAATACA	CCGATCAGGAATGACCGTAAG
*ACSL4*	GCGTTCCTCCAAGTAGACCAA	GCCTGTCATTCCAGCAATCAA
*FASN*	CCGTGTGACCGCCATCTAT	TGCTGTCGTCTGTAGTCTTGA
*ACAA2*	GGACTTCTCTGCCACCGATT	ACATTGCCCACGATGACACT
*HADH*	GGAGAACCTGAAGCTGAAGAAC	CTTGTCTGGTGGTGGCATTG
*PPT1*	GTTCTCACATCTGCGACTTCAT	CTGCCAAGAAGATGCTGTAGTT
*ELOVL5*	CCTCTGGTGGTACTACTTCTCC	GGTAGCGTGGTGGTAGACAT
*ELOV6*	ACGAGAACGAAGCCATCCA	ATCAGATGCCGACCACCAA
*HSD17B12*	GCCTCGTACTCGCTCTTCA	CCATCAGTGCCACCTGTAAC
*HACD2*	CGGCGTACCTGGTCATCTAC	GGCTCCTGTCTGGAAGAACTT
*ACOT2*	TGAAGAAGCCGTGAACTACCT	GGAGCCATTGATGACCACAG
*ACAT2*	TGCTGCTGTGGTCCTTATGA	TATGGCTGGAATTGGTCCTACT
*ACAA1*	GCTGAGATTGTGCCTGTGAC	GGTAGAGCCTCCATCCTTGAA
*ACOX1*	GGCGTGGAACTTGACTTCTG	CGGCTCTGTCTTGAATCTTGG
*ACADM*	ATTACCGAAGAGTTGGCGTATG	TCATTGGCTGCTCCGTCAT
*CPT1B*	AAGCACACCAGGCAGTAGC	TCCAGGAGTTGATTCCAGACAG
*GK*	CCTCCTGACAACCGTAGCA	TCTCTTAGCCAGCGGATTACA
*AGPAT2*	GTGCTCTGCCTGTCCTTCT	CTTCTGTCCGCTGACCTCAA
*DGAT1*	GCTATCCAGACAACCTGACCTA	GCATCTCAAGAACTCGTCGTAG
*LPL*	ACAAGGTCAGAGCCAAGAGAA	GTTGCTTGCCATCCTCAGTC
*FAS*	CTGGCTCACAGTTAAGAGTTCA	CAGGTTGGCATGGTTGACAG
*UCP1*	TGGAGGTGTGGCAGTGTTC	TCTGTGGTGGCTATAACTCTGT
*PPARGC1A*	TCGCTGCTCTTGAGAATGGA	TCGTCTGAGTTGGTATCTAGGT
*ADIPOQ*	GCTCTCCTGTTCCTCTTAATCC	ATGCCTGCCATCCAACCT
*SREBF1*	TCAACAACCAAGACAGTGACTT	GCCAGAGAAGCAGAAGAGAAG
*CEBPA*	ACAAGAACAGCAACGAGTACC	GGTCATTGTCACTGGTCAACT
*SCD1*	GGTGATGTTCCAGAGGAGGTA	CCAGAGTGTATCGCAAGAAGG
*FADS2*	TGAAGAAGACTGCTGAGGACAT	GTGCCGAAGTACGAGAGGAT

## Data Availability

Data contained within the article are included in the text, further inquiries can be directed to the corresponding author.
